# Supplementary figures and images for: Continuous Cropping Inhibits Photosynthesis of Polygonatum odoratum
Source: Plants (Basel). 2023 Sep 25;12(19):3374. doi: 10.3390/plants12193374 (PMC10574191; doi:10.3390/plants12193374)

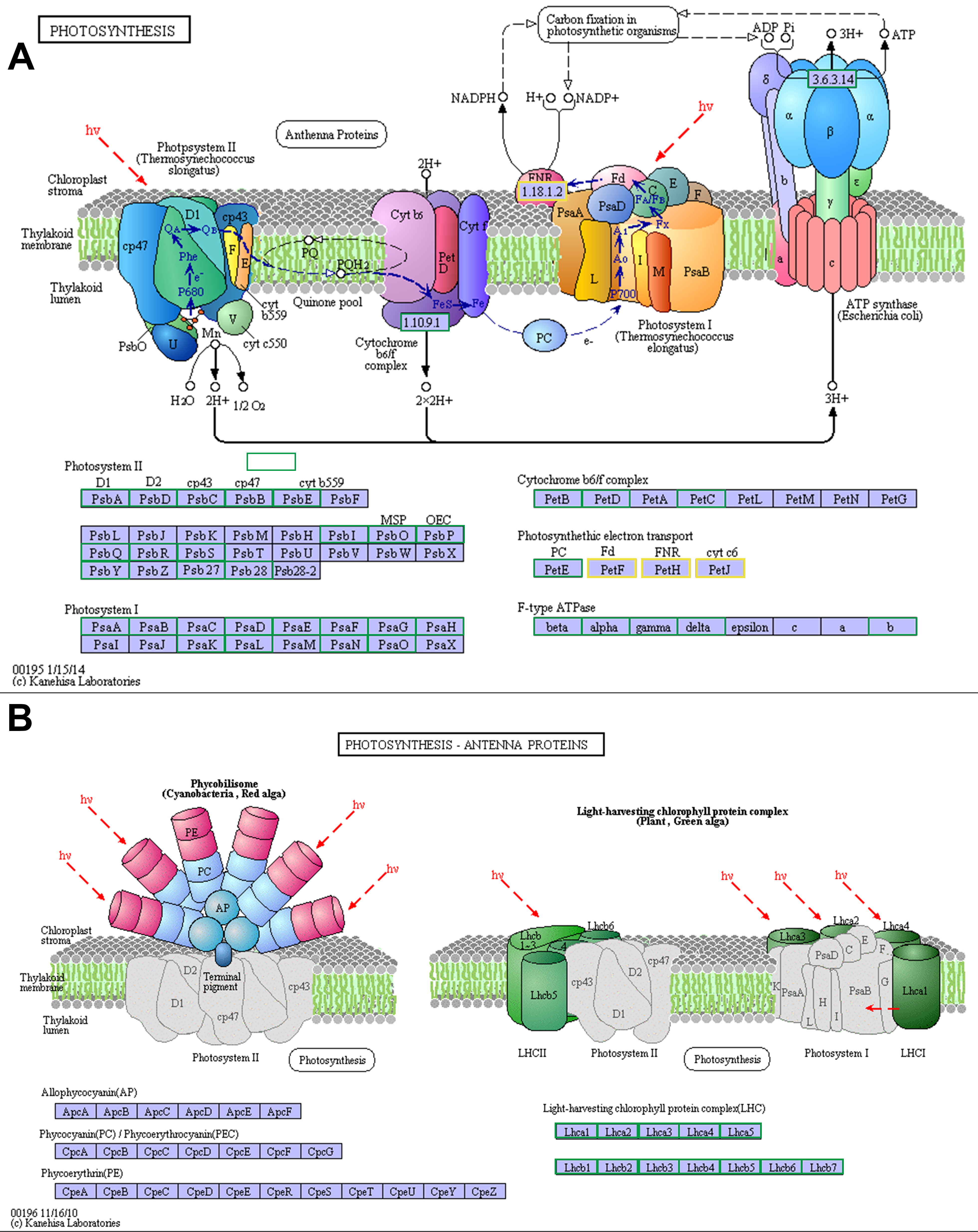

Supplement: Supplementary file 1 [file plants-12-03374-s001.zip › Figure S1.tif]
